# Supplementary material for: Generating high-quality plant and fish reference genomes from field-collected specimens by optimizing preservation
Source: Commun Biol. 2023 Dec 9;6:1246. doi: 10.1038/s42003-023-05615-2 (PMC10710401; doi:10.1038/s42003-023-05615-2)
Supplement: Supplementary file 2 — Supplementary Information [file 42003_2023_5615_MOESM2_ESM.pdf]

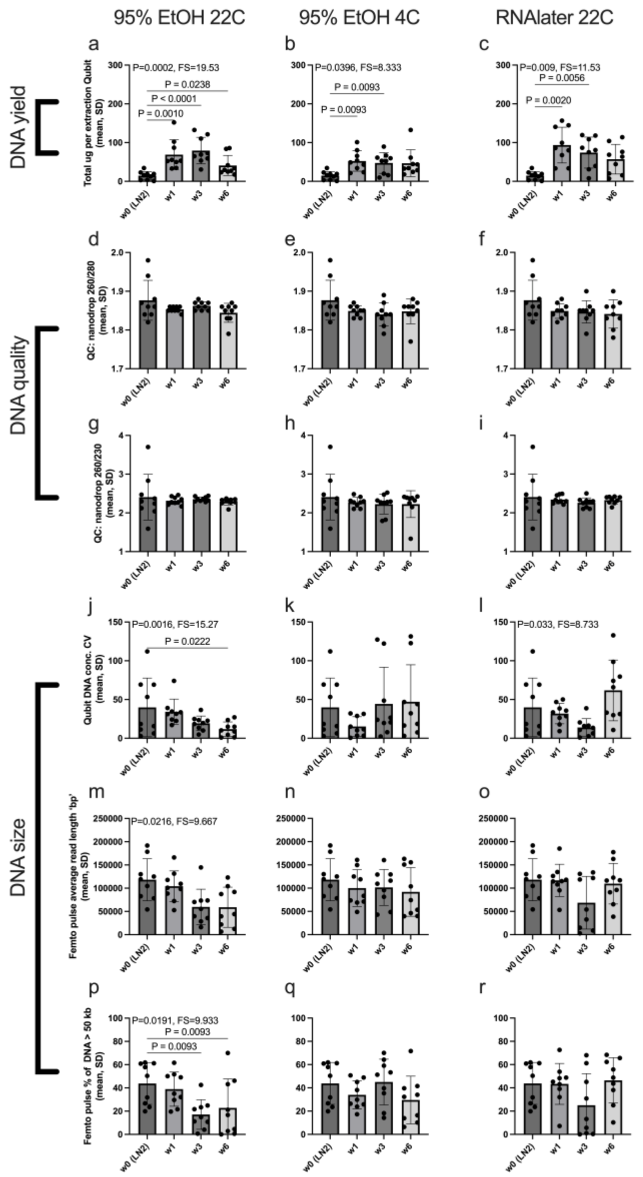

Supplemental Figure 1. Impacts of sample storage buffer, temperature, and time on fish DNA quality. Variation of DNA yield (total ug) as measured by Qubit BR kit amongst samples stored in a) 95% EtOH 22°C, b) 95% EtOH 4°C, and c) RNAlater 22°C. Variation in DNA quality 260/280 as measured by Nanodrop amongst samples stored in d) 95% EtOH 22°C, e) 95% EtOH 4°C, and f) RNAlater 22°C. Variation in DNA quality 260/230 as measured by Nanodrop amongst samples stored in g) 95% EtOH 22°C, h) 95% EtOH 4°C, and i) RNAlater 22°C. Variation in DNA fragment size as measured by the coefficient of variation of Qubit readings from top, middle, and bottom of tube across j) 95% EtOH 22°C, k) 95% EtOH 4°C, and l) RNAlater 22°C. Variation in mean DNA fragment size (bp) as measured by femto pulse across m) 95% EtOH 22°C, n) 95% EtOH 4°C, and o) RNAlater 22°C. Variation in DNA fragment sizes greater than 50kb as measured by femto pulse across p) 95% EtOH 22°C, q) 95% EtOH 4°C, and r) RNAlater 22°C.

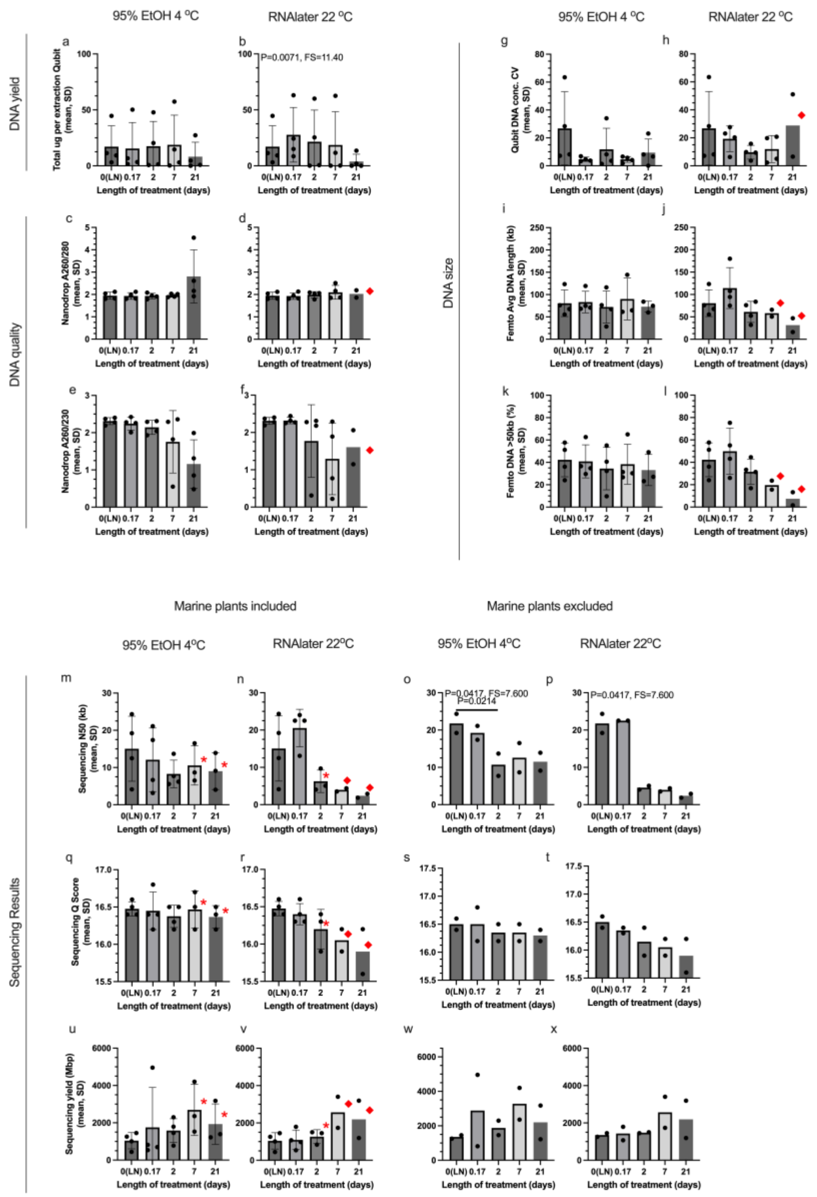

Supplemental Figure 2. Impacts of sample storage buffer, temperature, and time on plant DNA quality and sequencing. DNA samples from 4 plants were sequenced on Oxford Nanopore Promethion LSK114. Given the low yields for some later marine plant (Z. marina and P. torreyi) time points, the sequencing statistics are presented with and without these plants present, and statistical analysis run on the terrestrial plants only. Variation of DNA yield (total ug) as measured by Qubit BR kit amongst samples stored in a) 95% ETOH at 4°C and b) RNAlater at 22°C. Variation in DNA quality 260/280 as measured by Nanodrop amongst samples stored in c) 95% ETOH at 4°C and d) RNAlater at 22°C. Variation in DNA quality 260/230 as measured by Nanodrop amongst samples stored in e) 95% ETOH at 4°C and f) RNAlater at 22°C. Variation in DNA fragment size as measured by the coefficient of variation of Qubit readings from top, middle, and bottom of tube across g) 95% ETOH at 4°C and h) RNAlater at 22°C. Variation in mean DNA fragment size (bp) as measured by femto pulse across i) 95% ETOH at 4°C and j) RNAlater at 22°C. Variation in DNA fragment sizes greater than 50kb as measured by femto pulse across k) 95% ETOH at 4°C and l) RNA later at 22°C. Variation in sequencing results as measured by NanoPlot output for N50 across all sequenced samples for m) 95% ETOH at 4°C and n) RNAlater at 22°C, and across only terrestrial samples for o) 95% ETOH at 4°C and p) RNAlater at 22°C. NanoPlot mean read quality across all sequenced samples for q) 95% ETOH at 4C and r) RNAlater at 22°C, and across only terrestrial samples for s) 95% ETOH at 4°C and t) RNAlater at 22°C. NanoPlot total yield across all sequenced samples for u) 95% ETOH at 4°C and v) RNAlater at 22°C, and across only terrestrial samples for w) 95% ETOH at 4°C and x) RNAlater at 22°C. * indicates the time point contains a single marine plant sample below QC detection limit or omitted from sequencing, and the time point is omitted from the statistical analysis in that graph, ♦ indicates the time point contains two marine plant samples below QC detection limit or omitted from sequencing, and the time point is omitted from the statistical analysis in that graph

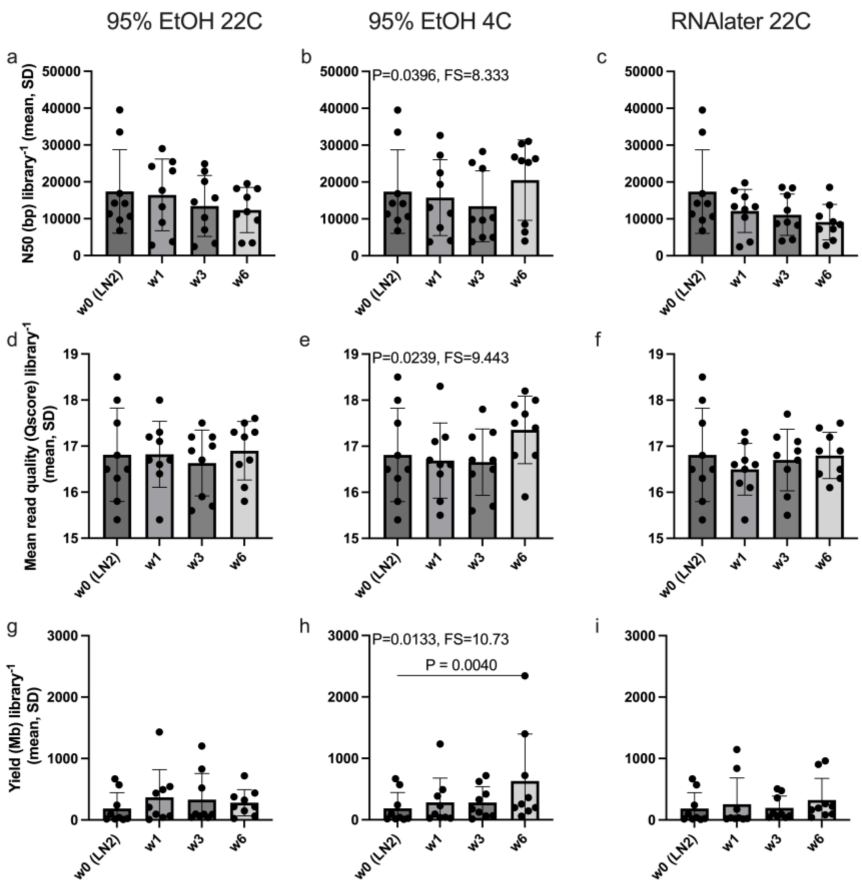

Supplemental Figure 3. Impacts of sample storage buffer, temperature, and time on fish sequencing. DNA samples from 9 fish (1 fish excluded) were sequenced on Oxford Nanopore Promethion LSK114. Variation in sequencing results as measured by NanoPlot output for N50 across a) 95% EtOH 22°C, b) 95% EtOH 4°C, and c) RNAlater 22°C; mean read quality across d) 95% EtOH 22°C, e) 95% EtOH 4°C and f) RNAlater 22°C; and total yield across g) 95% EtOH 22°C, h) 95% EtOH 4°C, and i) RNAlater 22°C

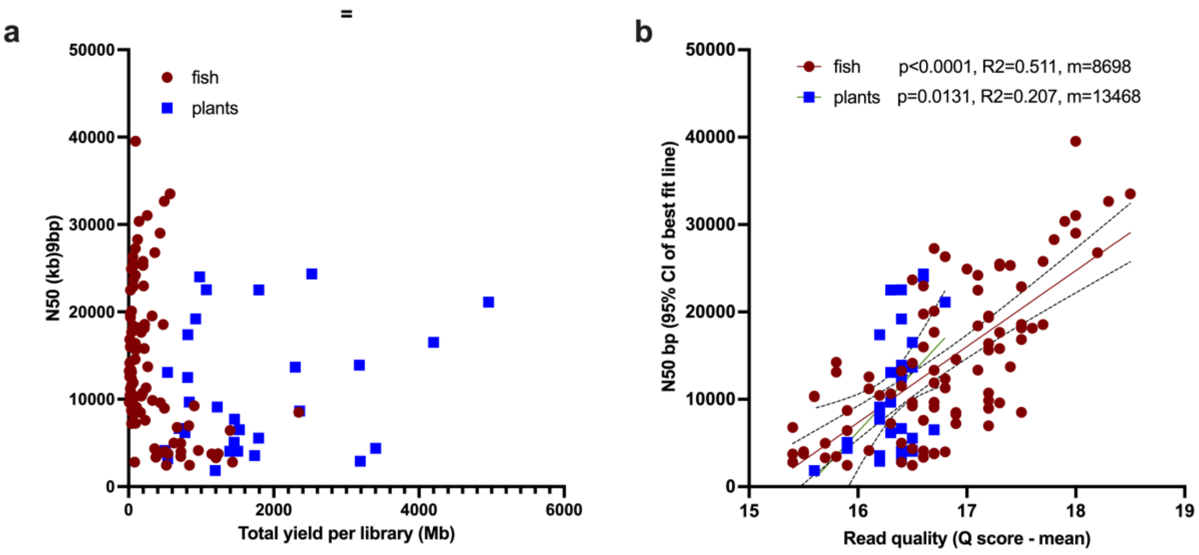

Supplemental Figure 4. Impacts of sequencing metrics on sequencing read N50. a) total library yield and b) mean read quality per library on N50 scores. All DNA from 90 fish samples and 29 plant samples were run on two separate sequencing runs (R000-469, and R000-471, respectively) using the LSK114 native barcoding kit. Average read quality, total yield, and N50 scores were generated from NanoPlot. Linear regression with 95% CI shown in panel b.

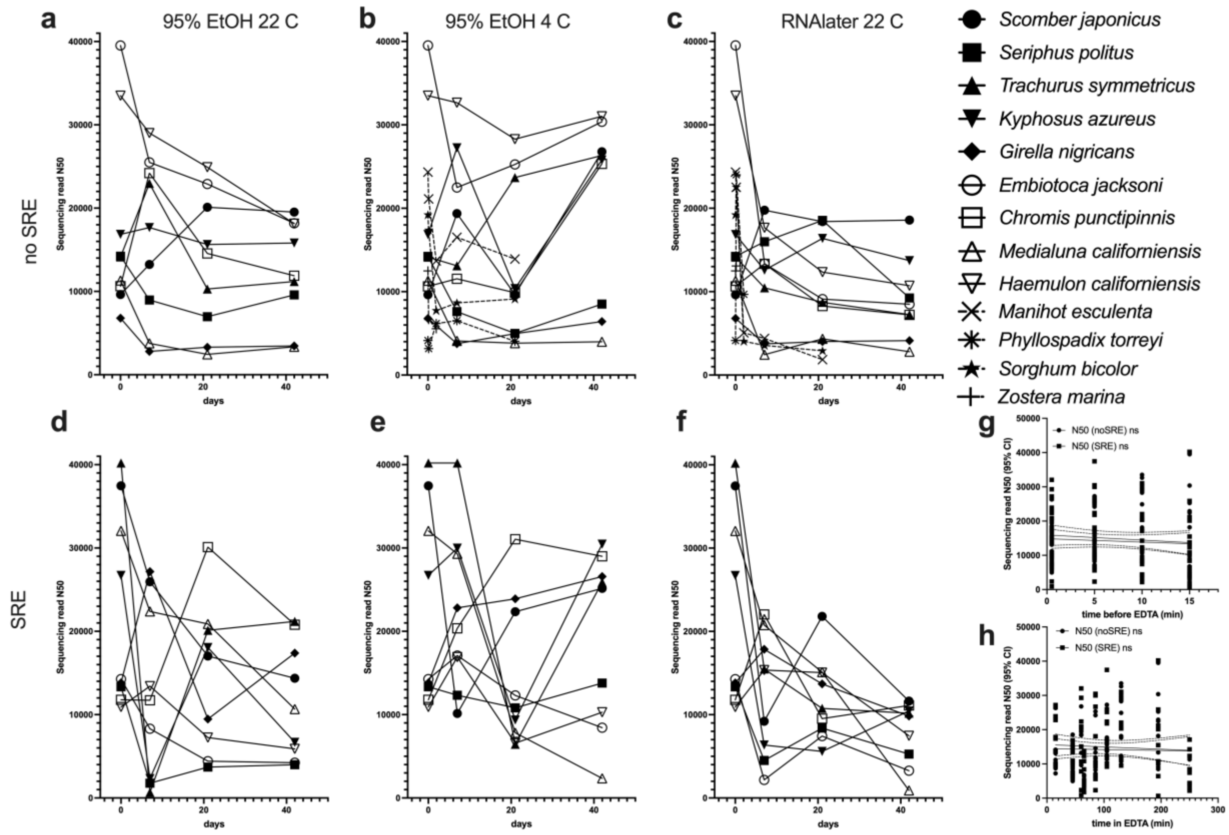

Supplemental Figure 5. Variation of sequencing N50 across fish samples as a result of storage buffer, time, and use of short read eliminator. Sequencing N50 results of untreated DNA from samples stored in a) 95% EtOH 22°C (9 fish species), b) 95% EtOH 4°C (9 fish and 4 plant species), and c) RNAlater 22°C (9 fish and 4 plant species). Plants are indicated by a dashed line while fish have solid line. Sequencing N50 results of DNA processed through the ONT SRE kit d) 95% EtOH 22 oC (9 fish species), e) 95% EtOH 4°C (9 fish species), and f) RNAlater 22°C (9 fish species). Impacts of blood collection methods also compared to sequencing N50. Specifically, g) impact of time that blood remained inside fish prior to being dispensed into an EDTA tube on sequencing N50 (30 seconds to 15 minutes) and h) impact of time in which EDTA blood tube was incubated on ice prior to being aliquoted to storage buffers (15 minutes to 4 hr 10 min). (g-h) Statistical significance tested with linear model and Spearman correlation.

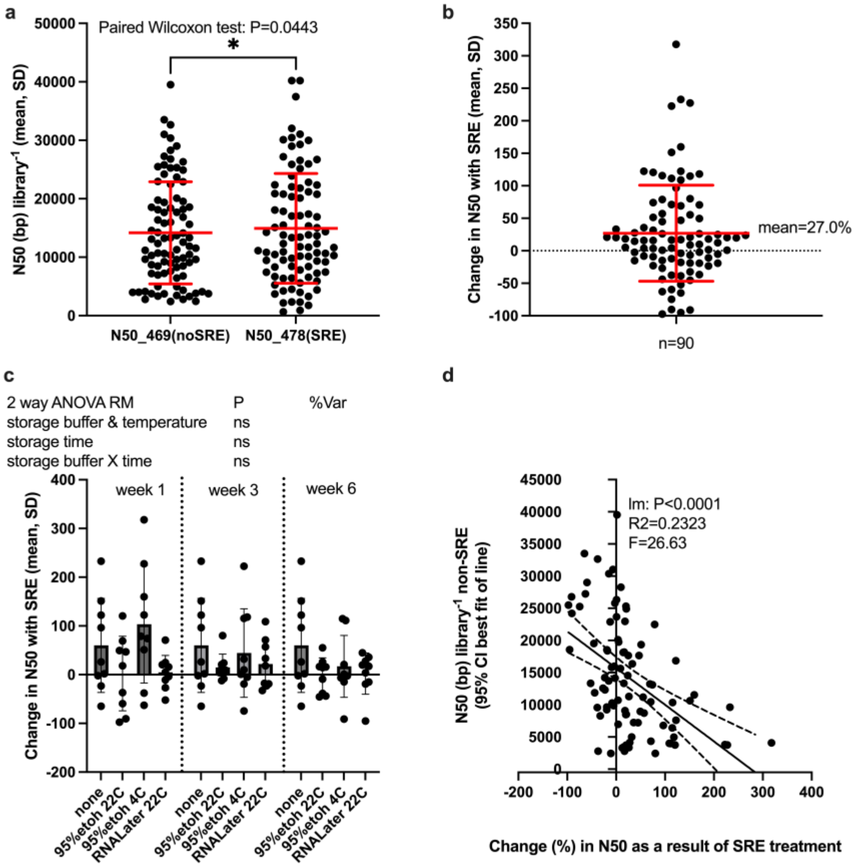

Supplemental Figure 6. Impacts of short read exclusion kit on sequencing performance as measured by sequencing read N50 generated by NanoPlot. a) All 90 DNA fish samples processed in two sequencing runs with and without SRE with pairwise comparison of N50 (Paired Wilcoxon test; P<0.05 *). b) Percent change of N50 as a result of using the SRE kit (positive indicates that the N50 increased with using SRE), c) comparison of sample storage groups on benefit of SRE, and d) impacts of change in N50 as a result of using SRE on the original N50 value (preSRE). Linear model for assessment.

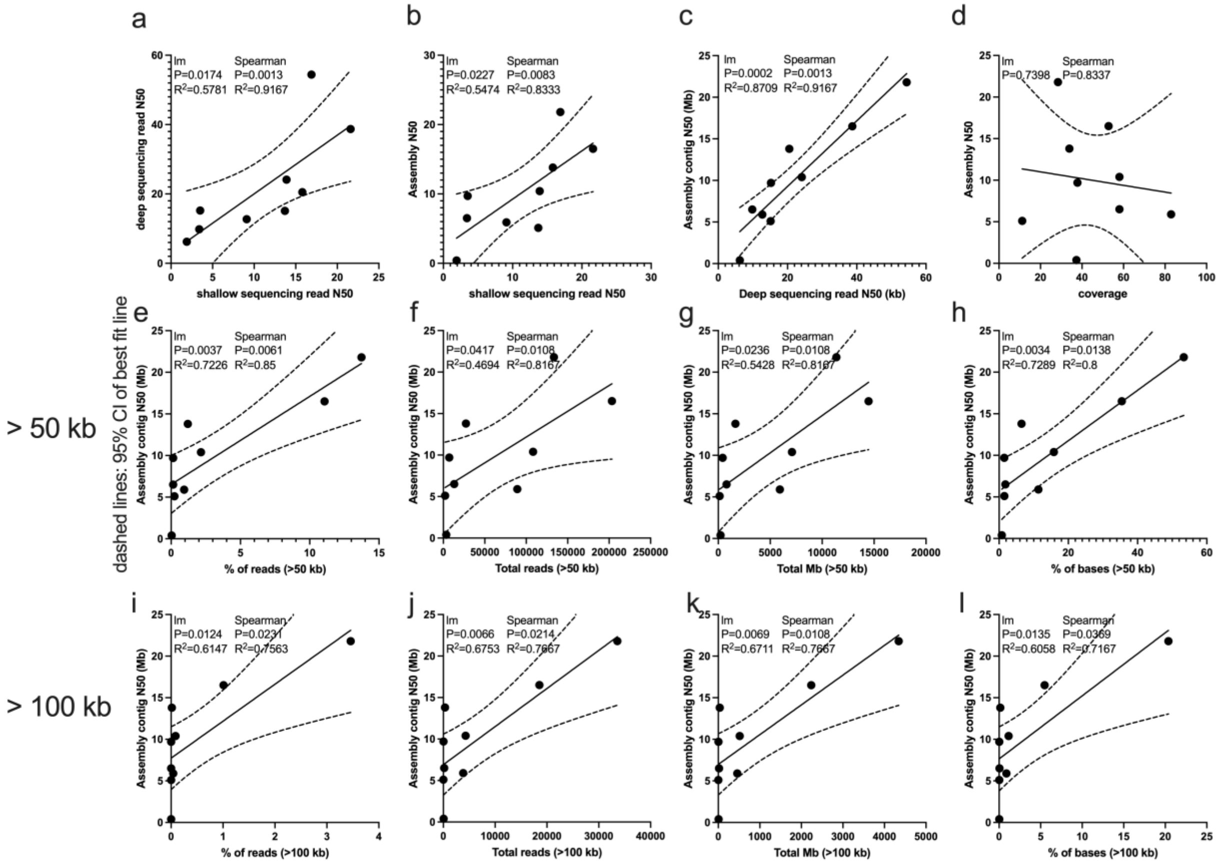

Supplemental Figure 7. Sequencing metrics predictive of genome assembly contig N50. a) Association between shallow sequencing read N50 and deep sequencing N50 and b) assembly contig N50. c) Comparison between deep sequencing read N50 with assembly contig N50. d) Association (or lack of) between sequencing coverage and assembly contig N50. For e-l, data files were subset to include only reads greater than 50 kb or 100 kb. e) The percent of total reads which were > 50 kb was compared to assembly contig N50. f) The total count of reads > 50 kb was compared to assembly contig N50. g) The total amount of bases in Mb of reads > 50 kb was compared to assembly contig N50. h) The percent of bases which had read lengths > 50 kb was compared to assembly contig N50. The same comparisons were computed for reads > 100 kb. Specifically, i) % of reads > 100 kb, j) total number of reads > 100 kb, k) total amount of bases in Mb, and l) percent of bases > 100 kb were compared to the assembly contig N50. Note, the assembly contig N50 is the assembly metric computed using the entire full sequencing run which includes all read lengths.

Supplementary Note 1: Fish Collection, Storage, and Processing SOP
How to collect and preserve samples from the field for later use in long-read sequencing
Goal: preserve high quality fish samples for whole genome sequencing using long-reads
Standard Operating Procedure
Version 1.0, jminich@salk.edu [fish], mamoore@ucsd.edu [plants], tmichael@salk.edu
Date: 2023-11-09

Materials needed:

- Syringe w/ Luer-Lok tip (3-10 ml) Becton Dickinson 5 ml [Cat # 309646]
- Needles (20-25 gauge, 20 G 1 1/2 works good) Becton Dickinson [Cat #305175]
- K2 EDTA 3.6 mg, BD vacutainer storage tube Becton Dickinson [Cat #367841]
- 100% Ethanol (EtOH)
- 1.5 or 2.0 ml tubes (e.g. eppendorf DNA Lobind tubes Cat# 022431021)
- NEB Monarch HMW kit (New England Biolabs, Ipswich, MA, Cat#T3050L)

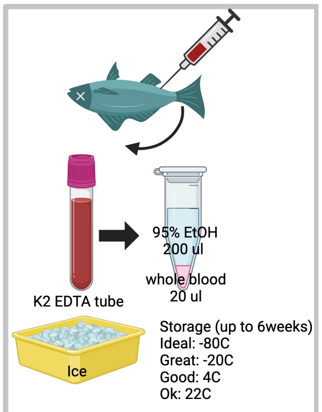

Fig 1. schematic of sample collection and storage

Sample collection and storage: (see Fig. 1)
1.Prepare syringe by attaching needle to syringe (using gloves)
2.Add ~ 200 ul ~95-100% EtOH to 1.5 mL tubes if using 20 ul of blood) (scale up as needed maintaining ratio)
3. Collect fish using rod and reel, spear, trawl, etc.
4. Hold fish upside down with tail towards the ground to allow blood to reach tail
5. For fish ~ > 10 g in size, collect blood with syringe (step 6)
For fish < 10 g in size, collect blood by any means necessary (e.g. cut off tail and try to obtain blood from caudal vein; cut gills and drip blood into a tube or weigh boat; stick needle directly into heart area)
6. Enter the caudal vein of the fish by inserting the needle at a 45 degree angle until you reach to backbone. Angle the syringe then at a 90 degree angle (perpendicular to fish) and begin drawing blood
7. Dispense blood into a K2 EDTA tube and mix by flicking or rotating up and down a few times. (Note, getting the blood into the EDTA tube as quick as possible is ideal as it will prevent or reduce clotting. The maximum amount of time we allowed fish to be dead for prior to putting blood into EDTA tubes was ~15 minutes in our study. We have not tested longer times.
8. Keep the blood filled K2 EDTA tube on ice if possible until transport back to the lab or until you are able to transfer to a 100% ethanol tube. (Note, our study had blood stored on ice in this way for between 15 min and up until ~ 4 hours and 10 minutes. We have not tested extended times other than one anecdotal experience of having fish blood stored in EDTA tubes for 48 hours on ice and obtaining good sequencing results, N50 > 50 kb). We have also not tested if ice is required)
9. Transfer 20 ul of whole blood to a 1.5 mL tube filled with 200 ul of 100% EtOH. If more blood is collected, scale accordingly (e.g. 100 ul blood + 1 mL 100% ethanol). Rotate several times and store on ice at 4C (ideal) or at room temperature 22C for up to 6 weeks. Although not tested, we suspect that storage in -20 or -80 C is the ideal condition if feasible.
DNA extraction (modifications):
10. Centrifuge the sample (20 ul of blood in 200 ul of 100% EtOH), at ~5000 RPM for 2 minutes.
11. Pipet off (remove) the ethanol storage solution and take the blood pellet into DNA extraction
12. Using the NEB Monarch kit "Fresh nucleated blood protocol", process up to 20 ul (can be as low as 5 ul, although we didn't test this)
13. For lysis, we used the highest setting of 2000 RPM. Note, you will likely obtain even longer DNA with a lower setting at the expense of less yield. We did not test this, but is worth considering for optimization
14. We eluted with 100 ul of buffer although may be better to increase to 200 ul (following protocol). We deviated from the protocol by not doing any pipet shearing of the DNA after elution.
15. Store gDNA in the fridge for at least 1 week before further processing (including sequencing). Do not freeze.

Please cite: Minich, J. J.*, Moore, M. L.*, Allsing, N. A., Aylward A., Murray, E. R., Tran, L., & Michael, T. P. (2023). Generating high quality plant and fish reference genomes from field collected specimens by optimizing preservation. Comms Bio.

Supplementary Note 2 – Collection, storage, and extraction of plants

Standard Operating Procedure
Goal: preserve high quality plant samples without -80C storage for whole genome sequencing using long- reads
Version 1.0, jminich@salk.edu [fish], mamoore@ucsd.edu [plants], tmichael@salk.edu
Date: 2023-08-25

DNA extraction steps adapted from ONT community protocol (https://community.nanoporetech.com/extraction_method_groups/plant-leaf-gDNA) with minimal alterations; steps written out here for ease of use.

Consumables:

- 100% Ethanol (EtOH)
- 100% Isopropanol (IPA)
- Tris(hydroxymethyl)aminomethane (Tris base)
- Sodium chloride (NaCl)
- Ethylenediaminetetraacetic acid sodium salt (EDTA)
- Cetyltrimethylammonium bromide (CTAB)
- Polyethylene glycol (PEG) 8000
- β-mercaptoethanol (BME)
- Chloroform
- RNase A, 100mg/mL (eg. QIAGEN Mat. #1007885)
- Nuclease-free water
- QIAGEN Blood and Cell Culture DNA Midi Kit (Cat. #13343)
- 50 mL Falcon tubes (e.g. Genesee Scientific Cat# 28-106
- 1.5 mL tubes (e.g. eppendorf DNA Lobind tubes Cat# 022431021)
- Kimwipes (Kimberly Clark Professional Cat# 34155)
- Liquid nitrogen (LN2)

Equipment:

- Scissors or small garden shears
- Mortar and pestle
- Stir plate
- Heat block or water bath
- Vortex
- Refrigerated centrifuge for spins up to 3,500 x g with 50mL

Sample collection and storage:

1. Prepare 95% EtOH (10-20 mL per sample) and place on ice in a cooler large enough to transport samples. If working with a large number of samples, consider pre-filling falcon tubes with 10-20 mL 95% EtOH prior to field sampling.
2. In the field, collect 1-5g young leaf tissue using clean scissors or shears. These will often be delicate, lighter green and lack wear and tear (breakage, pests, disease, etc). Non-woody stem tissue may also be used but has not been validated by this study. Remove any evident dirt or other surface contamination.
3. Cut leaves into ~1cm strips and add to a 50mL falcon tube. Immediately submerge in 95% EtOH.
4. Store on ice or at 4C for transport, up to 3 weeks. Though not tested, a -20 freezer may be used.

Day prior to DNA extraction:

5. In lab, prepare stock solutions necessary for lysis buffer: 1M Tris-HCl pH 9.5, 5M NaCl, 500mM EDTA
6. Prepare desired volume of Carlson lysis buffer (100 mM Tris-HCl, pH 9.5, 2% CTAB, 1.4 M NaCl, 1% PEG 8000, 20 mM EDTA) and mix overnight on a magnetic stirrer.

Lysis and first precipitation:

7. Pre-heat a heat block or water bath to 65°C and place in a fume hood.
8. For each extraction, transfer 20 ml of Carlson lysis buffer to a 50 ml Falcon tube.
9. In a fume hood, add 50uL BME and mix by vortexing. Pre-warm the solution to 65°C for ~30 minutes.
10. While the lysis buffer warms, pre-chill a clean mortar and pestle with LN2. Pour LN2 into the mortar and allow it to boil off at least twice before adding sample. Alternatively, the mortar may be pre- chilled in a -80C freezer.
11. Remove plant tissue from 95% EtOH and blot dry with a Kimwipe, or a clean paper towel. Discard EtOH from sample tube and allow tube to air dry.
12. Add sample tissue to the LN2 in the mortar and grind to a fine powder, around 5-10 minutes dependent on sample type. While grinding, continuously add LN2 to ensure that the sample stays cold. Once ground, proceed immediately into DNA extraction, or store sample in a -80 freezer.
13. Scoop 0.25-0.5 teaspoons ground tissue into the pre-warmed lysis buffer. Invert 5 times.
14. Add 40 μl of RNase A and vortex for 5 seconds.
15. Optional: If using a heat block with mixing, set the block (still at 65°C) to mixing at 300rpm for 5 minutes.
16. Incubate for 1 hour at 65°C. Invert 10 times every 15 minutes. At 30 minutes, add another 40 uL of RNase A, inverting 10 times to combine.
17. Allow the tubes to cool down to room temperature for 10 minutes.
18. Add 20 mL chloroform and vortex for two pulses of 5 seconds each.
19. Centrifuge the tubes at 3500 x g for 15 minutes at 4°C.
20. In a fume hood, transfer the top layer of lysate from each tube to a new 50 ml Falcon tube, without disturbing the interphase. The lysate layer should be 14-18mL of solution, but it is recommended to use wide-bore tips, transferring 1mL at a time. Tips can also be widened by cutting standard P1000 tips.
21. Add 0.7X volumes IPA. Invert 10 times. Incubate at -80°C for 15 minutes. Do not extend this incubation.
22. Centrifuge the sample at 3500 x g for 45 minutes at 4°C. If available, a fixed-angle centrifuge will create a pellet on the wall of the tube that has greater surface area for dissolution in step 15 (as compared to a conical pellet at the base of a falcon tube from a swinging bucket).
23. Discard the supernatant without disturbing the pellet. Use sterile wipes to absorb the liquid on the tube walls, being careful not to disturb the pellet.
24. To each pellet, add 10 mL G2 buffer, from the QIAGEN kit. Incubate at 50°C for 30-60 minutes, or until the pellet is dissolved. Swirl the pellet to mix but do not try to pipette or vortex.

Column cleanup:

25. Equilibrate a QIAGEN Genomic-tip 100/G column with 4 mL of Buffer QBT.
26. Pour the DNA in G2 buffer through the equilibrated column and allow it to flow through with just gravity.
27. Once all the lysate has passed through, wash the column with 8 mL of Buffer QC.
28. Repeat the wash with another 8 ml of Buffer QC.
29. Place the column over a clean 50 mL Falcon tube, and elute the genomic DNA with 5 mL of Buffer QF, pre-warmed to 55°C.
30. Allow the eluate to cool down to room temperature.

31. Add 3.5 mL IPA to the eluted DNA and mix by inverting the tube 10 times.
32. Incubate the tube at -20°C for at least 3 hours, or overnight.

Final precipitation:

33. Centrifuge the tube at 3500 x g for 45 minutes at 4°C.
34. Discard the supernatant without disturbing the pellet.
35. Add 4 ml of ice-cold 70% EtOH to the pelleted DNA and invert the tube 10 times.
36. Centrifuge at 3500 x g for 10 minutes at 4°C. If using a swinging bucket centrifuge the DNA will pellet at the base of the tube and be easy to locate and resuspend. If using a fixed angle, mark the side of the tube that faces outwards in order to locate the pellet for washes and elution.
37. Discard the supernatant without disturbing the pellet. Use sterile wipes to dry the tube walls, being careful not to disturb the pellet.
38. Resuspend the DNA in 100 µL of TE buffer and incubate at room temperature, typically overnight.
39. Transfer the DNA into a nuclease-free 1.5 mL tube (DNA LoBind tube preferred) using a wide-bore tip, and store at 4°C. Often, waiting a further 48 hours before quantifying on Nanodrop and Qubit will allow the DNA to further relax and yield the most accurate results

Please cite: Minich, J. J.*, Moore, M. L.*, Allsing, N. A., Murray, E. R., Tran, L., & Michael, T. P. (2023). Generating high quality plant and fish reference genomes from field collected specimens by optimizing preservation. Comms Bio.
