# Supplementary material for: Generating high-quality plant and fish reference genomes from field-collected specimens by optimizing preservation
Source: Commun Biol. 2023 Dec 9;6:1246. doi: 10.1038/s42003-023-05615-2 (PMC10710401; doi:10.1038/s42003-023-05615-2)
Supplement: Supplementary file 4 — Reporting Summary [file 42003_2023_5615_MOESM4_ESM.pdf]

nature portfolio

Corresponding author(s): Dr. Todd Michael
Last updated by author(s): Nov 7, 2023

Reporting Summary

Nature Portfolio wishes to improve the reproducibility of the work that we publish. This form provides structure for consistency and transparency in reporting. For further information on Nature Portfolio policies, see our Editorial Policies and the Editorial Policy Checklist.

Statistics

For all statistical analyses, confirm that the following items are present in the figure legend, table legend, main text, or Methods section.

n/a	Confirmed	
☐	☒	The exact sample size (*n*) for each experimental group/condition, given as a discrete number and unit of measurement
☐	☒	A statement on whether measurements were taken from distinct samples or whether the same sample was measured repeatedly
☐	☒	The statistical test(s) used AND whether they are one- or two-sided<br>*Only common tests should be described solely by name; describe more complex techniques in the Methods section.*
☐	☒	A description of all covariates tested
☐	☒	A description of any assumptions or corrections, such as tests of normality and adjustment for multiple comparisons
☐	☒	A full description of the statistical parameters including central tendency (e.g. means) or other basic estimates (e.g. regression coefficient) AND variation (e.g. standard deviation) or associated estimates of uncertainty (e.g. confidence intervals)
☐	☒	For null hypothesis testing, the test statistic (e.g. *F*, *t*, *r*) with confidence intervals, effect sizes, degrees of freedom and *P* value noted<br>*Give P values as exact values whenever suitable.*
☒	☐	For Bayesian analysis, information on the choice of priors and Markov chain Monte Carlo settings
☒	☐	For hierarchical and complex designs, identification of the appropriate level for tests and full reporting of outcomes
☒	☐	Estimates of effect sizes (e.g. Cohen's *d*, Pearson's *r*), indicating how they were calculated

Our web collection on statistics for biologists contains articles on many of the points above.

Software and code

Policy information about availability of computer code

Data collection	Nanopore shallow sequencing: Promethion 10.4.1 flow cell with MinKnow version 22.1 with HAC basecalling<br>Nanopore deep sequencing: Promethion 10.4.1 flow cell with MinKnow version 22.1 with SUP basecalling<br>Methylation basecalling and data generation: ONT basecalling software Dorado version 0.2.4, SUP basecalling with R10.4.1 and 260bps translocation speed<br>Sequencing QC: Nanoplot 1.40.0, Genomescope 2.0, BUSCO 5.4.3, Meryl 1.3, Merqury 1.3<br>Assemblies generated using FlyE version 2.9 and Racon 1.4.20
Data analysis	Statistical analyses and figures made either using R or Prism version 9.4.1 with a paid license.

For manuscripts utilizing custom algorithms or software that are central to the research but not yet described in published literature, software must be made available to editors and reviewers. We strongly encourage code deposition in a community repository (e.g. GitHub). See the Nature Portfolio guidelines for submitting code & software for further information.

Data

Policy information about availability of data

All manuscripts must include a data availability statement. This statement should provide the following information, where applicable:

- Accession codes, unique identifiers, or web links for publicly available datasets
- A description of any restrictions on data availability
- For clinical datasets or third party data, please ensure that the statement adheres to our policy

All DNA QC and shallow sequencing QC data are represented in Supplemental Data 2. Sequencing data is publicly available (PRJNA971989). We've included two standard operating procedures (SOPs) for sample collection, storage, and extraction as supplemental data. This includes one for fish (Supplemental Data SOP Fish) and one for plants (Supplemental Data SOP Plant).

Research involving human participants, their data, or biological material

Policy information about studies with human participants or human data. See also policy information about sex, gender (identity/presentation), and sexual orientation and race, ethnicity and racism.

Reporting on sex and gender	*Use the terms sex (biological attribute) and gender (shaped by social and cultural circumstances) carefully in order to avoid confusing both terms. Indicate if findings apply to only one sex or gender; describe whether sex and gender were considered in study design; whether sex and/or gender was determined based on self-reporting or assigned and methods used. Provide in the source data disaggregated sex and gender data, where this information has been collected, and if consent has been obtained for sharing of individual-level data; provide overall numbers in this Reporting Summary. Please state if this information has not been collected. Report sex- and gender-based analyses where performed, justify reasons for lack of sex- and gender-based analysis.*
Reporting on race, ethnicity, or other socially relevant groupings	*Please specify the socially constructed or socially relevant categorization variable(s) used in your manuscript and explain why they were used. Please note that such variables should not be used as proxies for other socially constructed/relevant variables (for example, race or ethnicity should not be used as a proxy for socioeconomic status). Provide clear definitions of the relevant terms used, how they were provided (by the participants/respondents, the researchers, or third parties), and the method(s) used to classify people into the different categories (e.g. self-report, census or administrative data, social media data, etc.) Please provide details about how you controlled for confounding variables in your analyses.*
Population characteristics	*Describe the covariate-relevant population characteristics of the human research participants (e.g. age, genotypic information, past and current diagnosis and treatment categories). If you filled out the behavioural & social sciences study design questions and have nothing to add here, write "See above."*
Recruitment	*Describe how participants were recruited. Outline any potential self-selection bias or other biases that may be present and how these are likely to impact results.*
Ethics oversight	*Identify the organization(s) that approved the study protocol.*

Note that full information on the approval of the study protocol must also be provided in the manuscript.

Field-specific reporting

Please select the one below that is the best fit for your research. If you are not sure, read the appropriate sections before making your selection.

☒ Life sciences ☐ Behavioural & social sciences ☐ Ecological, evolutionary & environmental sciences

For a reference copy of the document with all sections, see nature.com/documents/nr-reporting-summary-flat.pdf

Life sciences study design

All studies must disclose on these points even when the disclosure is negative.

Sample size	The number of species present in the study was determined by balancing reproducibility with extraction time (~3 hours for fish, ~10 hours for plant) and sequencing costs. We believe the sample size sufficient to describe marine fish blood due to consistent results, and relatively sufficient for plant leaf tissue where results were less consistent across species. Further studies using nucleated blood from other vertebrates, or with plant tissues beyond leaf, will be necessary to extend this method beyond the tested sample types. But, the results are encouraging.
Data exclusions	We removed one fish (Halichoeres semicintus) from the study due to insufficient sample quantity. Additionally, 7 plant samples (of 36 samples) were excluded from sequencing as they fell below the threshold for library preparation. These exclusions are specified at the beginning of the Results and Discussion section, and in the relevant plant supplementary figure 2 where it affected statistical analysis.
Replication	We chose to include 10 fish (9 with H. semicintus exclusion) and 4 plants representing separate species as replicates. This has the benefit of demonstrating reproducibility across multiple species, while acknowledging the drawback of not including an additional level of replication at the species level, which would've been cost-prohibitive.

Randomization	This is not largely relevant, as all samples were subjected to the same treatments. In plants, tissue age extracted at each storage time point was normalized through randomization by cutting each sample for storage into ~1cm segments and aggregating these in buffer or solvent so that no single time point had a disproportionate amount of younger or older tissue to skew the extraction outcome.
Blinding	n/a

Reporting for specific materials, systems and methods

We require information from authors about some types of materials, experimental systems and methods used in many studies. Here, indicate whether each material, system or method listed is relevant to your study. If you are not sure if a list item applies to your research, read the appropriate section before selecting a response.

Materials & experimental systems

n/a	Involved in the study	
☒	☐	Antibodies
☒	☐	Eukaryotic cell lines
☒	☐	Palaeontology and archaeology
☐	☒	Animals and other organisms
☒	☐	Clinical data
☒	☐	Dual use research of concern
☐	☒	Plants

Methods

n/a	Involved in the study	
☒	☐	ChIP-seq
☒	☐	Flow cytometry
☒	☐	MRI-based neuroimaging

Animals and other research organisms

Policy information about studies involving animals; ARRIVE guidelines recommended for reporting animal research, and Sex and Gender in Research

Laboratory animals	This study did not use laboratory animals
Wild animals	This study used a total of 9 single animals from 9 different fish species. These fish were donated from local fishers. Tissue processing is covered under IACUC Animal Use Protocol S12219. Details of this are also in the manuscript.
Reporting on sex	All fish were juvenile (sexually immature), thus sex could not be deteremined.
Field-collected samples	Field collected samples were euthanized in the field by the fishers and then handed off to the researchers. Blood samples were collected in the field on the spot.
Ethics oversight	UCSD IACUC protocol S12219 gives approval for fish collection, housing, husbandry, and euthanizing. For this paper, we did not have to do most of this as it was already done but still the protocol covers it either way.

Note that full information on the approval of the study protocol must also be provided in the manuscript.

Plants

Seed stocks	NA
Novel plant genotypes	NA
Authentication	NA
